# *Chlamydia pneumoniae* Infection in Mice Induces Chronic Lung Inflammation, iBALT Formation, and Fibrosis

**DOI:** 10.1371/journal.pone.0077447

**Published:** 2013-10-25

**Authors:** Madhulika Jupelli, Kenichi Shimada, Norika Chiba, Anatoly Slepenkin, Randa Alsabeh, Heather D. Jones, Ellena Peterson, Shuang Chen, Moshe Arditi, Timothy R. Crother

**Affiliations:** 1 Division of Pediatric Infectious Diseases, Department of Pediatrics, Cedars-Sinai Medical Center, Los Angeles, California, United States of America; 2 Department of Pathology, University of California Irvine, Irvine, California, United States of America; 3 Department of Pathology and Laboratory Medicine, Cedars-Sinai Medical Center, University of California Los Angeles, Los Angeles, California, United States of America; 4 Division of Pulmonary and Critical Care Medicine, Department of Medicine, Cedars-Sinai Medical Center, Los Angeles, California, United States of America; French National Centre for Scientific Research, France

## Abstract

*Chlamydia pneumoniae* (CP) lung infection can induce chronic lung inflammation and is associated with not only acute asthma but also COPD exacerbations. However, in mouse models of CP infection, most studies have investigated specifically the acute phase of the infection and not the longer-term chronic changes in the lungs. We infected C57BL/6 mice with 5×10^5^ CP intratracheally and monitored inflammation, cellular infiltrates and cytokine levels over time to investigate the chronic inflammatory lung changes. While bacteria numbers declined by day 28, macrophage numbers remained high through day 35. Immune cell clusters were detected as early as day 14 and persisted through day 35, and stained positive for B, T, and follicular dendritic cells, indicating these clusters were inducible bronchus associated lymphoid tissues (iBALTs). Classically activated inflammatory M1 macrophages were the predominant subtype early on while alternatively activated M2 macrophages increased later during infection. Adoptive transfer of M1 but not M2 macrophages intratracheally 1 week after infection resulted in greater lung inflammation, severe fibrosis, and increased numbers of iBALTS 35 days after infection. In summary, we show that CP lung infection in mice induces chronic inflammatory changes including iBALT formations as well as fibrosis. These observations suggest that the M1 macrophages, which are part of the normal response to clear acute *C. pneumoniae* lung infection, result in an enhanced acute response when present in excess numbers, with greater inflammation, tissue injury, and severe fibrosis.

## Introduction

*Chlamydia pneumoniae* (CP), a gram-negative obligate intracellular bacterial pathogen, is responsible for up to 10% of community acquired pneumonias and infects most people by 60 years old [1], [2]. It causes acute respiratory tract diseases such as pneumonia, sinusitis, and bronchitis, and is associated with development of chronic lung diseases such as asthma. It is also widely associated with exacerbations of chronic obstructive pulmonary disorder, where chronic inflammation is a hallmark feature [3]–[6]. Chronic CP infection was first associated with wheezing, asthmatic bronchitis and adult-onset asthma in 1991 [7]. Subsequent studies of bronchoalveolar lavage fluid from pediatric patients with severe chronic respiratory illnesses including asthma have demonstrated that over half had evidence of CP by direct organism identification [8]. Additionally, chronic CP infections were associated with other inflammatory diseases such as atherosclerosis [9], [10]. How CP infection might induce and/or exacerbate various inflammatory diseases such as atherosclerosis is unknown, although one could postulate a more direct involvement in lung disorders. CP infects various cell types such as monocytes, macrophages, neutrophils, epithelial cells, smooth-muscle cells, and endothelial cells [11]–[13]. While successful infection usually results in CP progeny and host cell death, the organism can often reside intracellularly for indefinite periods [14], potentially promoting chronic inflammation.

Pulmonary fibrosis can also be induced during serious pulmonary infections. CP infection in mice can lead to fibrosis in animals that are deficient in IL-1 signaling [15]. These mice have greater inflammation early on, which leads to the development of fibrosis later. Considering the association with CP lung infection and chronic inflammatory diseases, together with the prevalence of this infection in elderly, the effects of CP infection on the development of lung fibrosis may have important consequences.

Another aspect of lung infections is the development of inducible bronchus associated lymphoid tissue (iBALT). iBALTs are thought to provide a local source of adaptive immunity and aid in the prevention of reinfection [16]. However, there is still debate as it appears that under some circumstances the development of iBALTs also contributes to acceleration of lung pathologies [17]–[19]. To date, no one has identified iBALTs in mouse models of CP infection, however, there is some suggestion that they may form, as lymphoid cell accumulations have been noted both perivascularly and peribronchial, especially during more severe infections [20]–[22]. Thus many of the long-term aspects of CP pulmonary infection have not been studied carefully in mouse models.

Macrophages are one of the most important host innate immune cells and play a key role in sensing and controlling bacterial infections. Macrophages are heterogeneous in phenotype and exhibit plasticity in polarizing to adapt to different tissue environments. Two types of macrophages have been identified in infection and tissue repair, namely classically activated macrophages or inflammatory macrophages (M1) and alternatively activated macrophages (M2) [23], [24]. M1 macrophages, are associated with higher pro-inflammatory cytokine production, which initiates adaptive immune T cell responses and induce tissue inflammation by producing cytokines and proinflammatory mediators such as iNOS, IL-12, IL-23, and TNF-alpha [25], [26]. Although M1 inflammatory macrophages effectively control pathogen invasion, prolonged inflammatory cytokine production induced by excessive M1 macrophages may lead to persistent inflammation as a result of excessive Th1 or Th17 responses, and can also impede the wound healing and repair processes, and induce tissue damage [27], [28]. M2 macrophages predominantly produce IL-10 and play a role in wound healing and tissue repair [29]. Resolving pathogen invasion and the initiation of tissue repair is associated with M2 macrophages through increased concentrations of growth factors and release of anti-inflammatory cytokines into the tissues [29].

In this study, we investigated the development of inflammatory pathology in pulmonary CP infection, not just during the acute infection, but also during the chronic phase of this infection. Wild type (WT) C57BL/6 mice infected intratracheally (i.t) with CP and followed for 35 days post infection. CP IFUs were substantially decreased by 28 days post infection and this roughly paralleled inflammatory lung pathology in these mice. Furthermore, these infected mice demonstrated clusters of T, B lymphocytes, follicular dendritic cells (fDCs)–iBALTs, with increased number of macrophages and cytokines in the lungs despite decreased bacterial titers. Macrophage populations early on were predominantly M1, while M2 were found in greater numbers at later time points. Adoptive transfer of M1 macrophages during infection demonstrated severe inflammation and fibrosis at later time points compared to adoptive transfer of M2 macrophages or control animals. This study shows that CP lung infection in mice induces chronic lung inflammation, iBALT formation, and fibrosis, which can be drastically enhanced by the presence of greater numbers of M1 macrophages.

## Results

### *C. pneumoniae* infection in mice induces chronic lung inflammation and the development of lymphoid aggregates in the lungs

**Figure 1:**
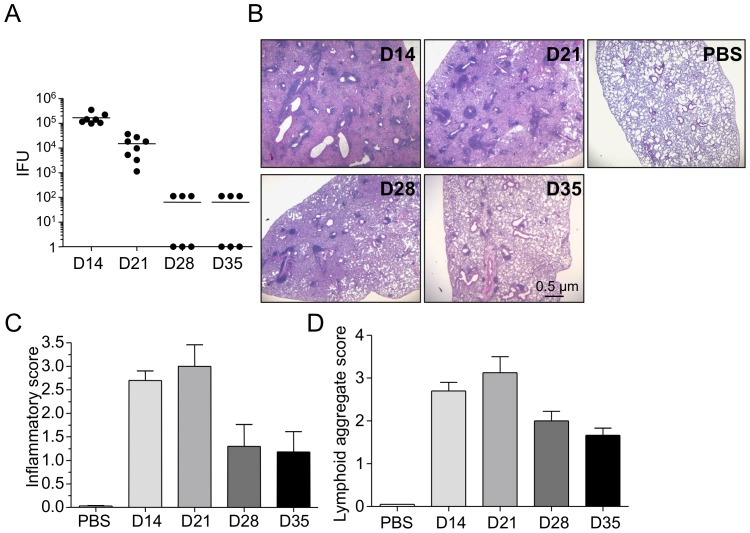
CP infection induced inflammation. C57BL/6 mice were infected intratracheally with 5x10^5^ IFU CP and sacrificed at various time points. A) CP bacterial burden. B) Representative H&E stained lung sections. C) Inflammatory score. D) Lymphoid aggregate score. Data for all experiments shown represent at least two independent experiments (pooled together).

To determine the kinetics of bacterial clearance in WT mice following *C. pneumoniae* infection, we infected WT C57/Bl6 mice i.t with 5×10^5^ inclusion forming units (IFU) of *C. pneumoniae*. Following CP infection, mice were allowed to rest, and lungs were harvested at multiple time points as shown in Fig 1, to determine bacterial numbers and degree of inflammatory pathology in the lungs. While most published CP infection studies focus on the first 1–2 weeks of infection (acute phase), here we investigated the chronic, longer-term responses to CP infection (days 14–35). As shown in Fig 1A, WT mice infected with CP demonstrated highest number of bacterial titers at day 14 post-infection, and they exhibited a temporal decrease in chlamydial IFUs in the subsequent time points, with the bacteria nearly undetectable by days 28–38 post infection. A similar trend was observed in development of inflammatory pathology in these mice at the observed time points. Mice infected with CP developed significant inflammatory lung pathology at day 14 post-infection (Figure 1B). Upon closer observation, these mice exhibited significant and patchy inflammatory cell clusters around the peribronchiolar and perivascular regions of the lung that could possibly be secondary lymphoid in origin, such as iBALTs (Figure 1B). This inflammatory pathology peaked at day 21 post-infection and showed a decreasing trend by day 28 and 35 post-infection. Inflammation score of the lung pathology showed gradually improving pneumonia, but we still observed large numbers of lymphoid aggregates by day 35 post-infection (Figure 1C and D).

### *Chlamydia pneumoniae* infection leads to iBALT formation in lungs of mice

**Figure 2:**
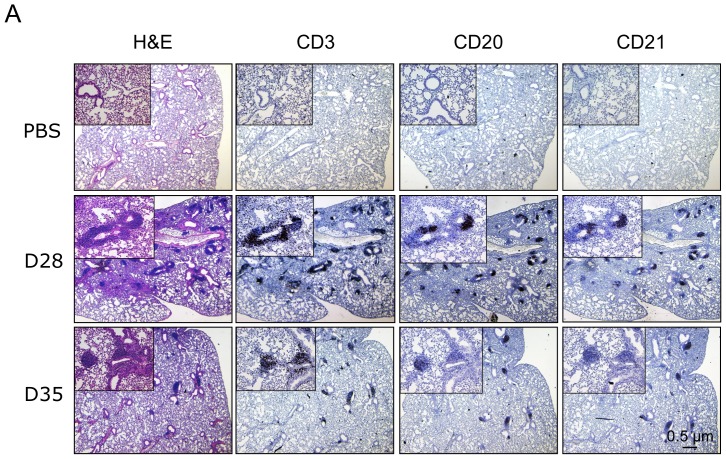
CP infection induces iBALT formation. A) Paraffin lung sections from CP infected animals were stained for the presence of T, B, and follicular dendritic cells using anti-CD3, CD20, and CD21 antibodies respectively.

Given the large clusters of immune infiltrates that we observed in the lungs after CP infection, we next wished to investigate whether these aggregates were indeed iBALTs, by immunostaining for the presence of B, T, and follicular dendritic cells (fDC). Using CD3, CD20, and CD21 as markers for B, T, and fDCs respectively, we observed that each of these cell types were present in large numbers in the immune cell clusters (Figure 2), identifying them as secondary lymphoid organs, also known as iBALTs in the lungs. There were more cells present at earlier time points (day 28 versus 35) suggesting the gradual reduction in iBALT maintenance with time.

### *Chlamydia pneumoniae* lung infection induces a persistent macrophage influx in the lungs throughout the course of infection

**Figure 3:**
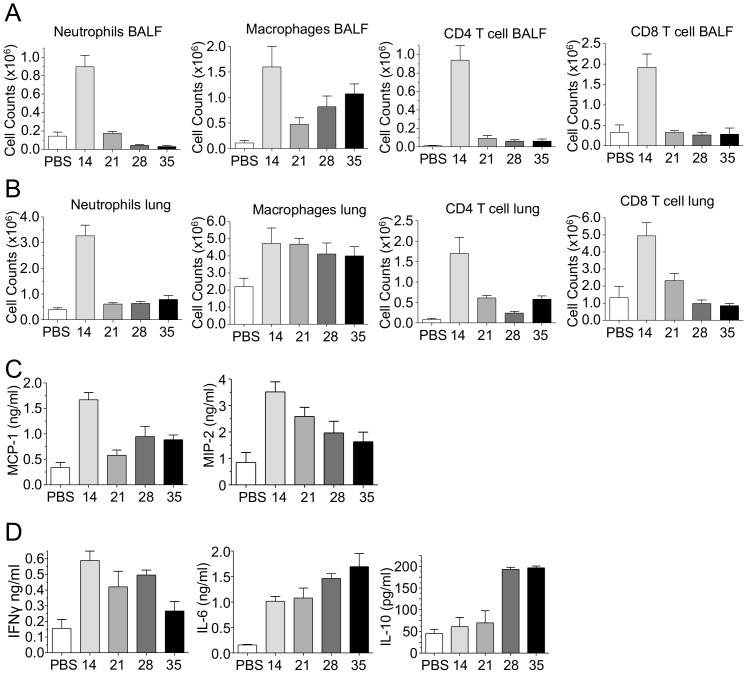
CP infection induced immune infiltrates and cytokines. Immune cell counts in the A) BALF and B) lung were assessed at various time points after CP infection by flow cytometry. C) Chemokine production in lung homogenates after infection. D) Cytokine production in lung homogenates after infection. Data for all experiments shown represent at least two independent experiments (pooled together).

We next examined the immune infiltrates into the lungs and airspaces using Flow cytometric analysis. Neutrophils, CD4, and CD8 T-cells peaked early during infection (day 14) and then rapidly decreased in numbers in both the BALF and single cell suspensions of the lungs (day 21) (Figure 3A and B), paralleling the reduction of CP titers. However, macrophage numbers in both the BALF and the lungs had altered temporal kinetics compared with the other immune cell types. In the airspaces, macrophages peaked early (day 14), and then dropped rapidly by day 21, only to see a slow increase in macrophages numbers over time (days 28–35) (Figure 3A). However, in the lung tissue, macrophages maintained a substantial presence with no reduction in cell numbers over time (Figure 3B). Since there were increasing numbers of macrophages over the long-term course of CP infection, despite the reduction in bacterial burden in the lungs, we assessed the amount of macrophages chemokines present in the lung. Both MCP-1 and MIP2 were present at day 14 after infection (Figure 3C). Both of these chemokines decreased over time, but neither returned to baseline levels and MIP2 showed a very gradual reduction. In addition to MCP-1 and MIP2, we also measured IFN-γ, IL-6 and IL-10 concentrations in the lung homogenates (Figure 3D). IFN-γ concentrations remained at increased levels early on and slowly decreased over time. IL-6, however, increased over time, indicating a source of continued inflammation, despite reduced CP IFUs (Figure 3D). Finally, IL-10, a suppressive cytokine, which was not present early on, was upregulated on days 28 and 35 post infection (Figure 3D), perhaps as a repair mechanism following the acute inflammation.

### M1 Macrophages promote CP infection-induced lung inflammation and iBALT formation

**Figure 4:**
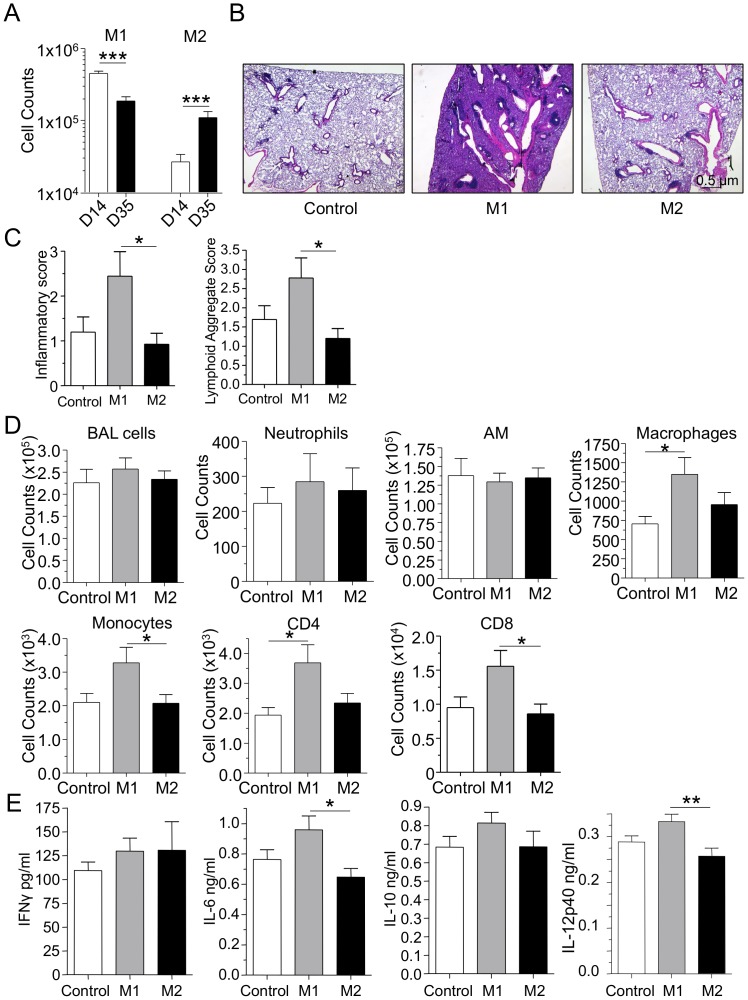
Adoptive transfer of M1 macrophages into CP infected mice induces severe long-term inflammation. A) M1 and M2 macrophage numbers in lung single cell suspensions after CP infection. B–E) 1×10^6^ M1 or M2 BMM were adoptively transferred into mice 7 days after CP infection (5×10^5^ IFU). Mice were sacrificed 35 days after infection. B) Representative H&E stained lung section. C) Inflammatory and lymphoid aggregate scores. D) BALF cell count and immune cell counts in the lung. E) Cytokines in lung homogenates. Data for all experiments shown represent at least two independent experiments (pooled together). *p<0.05, **p<0.01, ***p<0.001 (Student's t test or One-Way ANOVA).

Since we observed that macrophage numbers were increased throughout the course of CP infection, we next investigated the nature of these macrophages. Using iNOS and CD206 as markers for M1 versus M2 macrophages respectively, we found that M1 macrophages decreased over time from days 14 to 35, while M2 macrophages significantly increased over that same time span (Figure 4A). This observation coincided with the increase in IL-10 levels (Figure 3D) and suggested that M2 macrophages were increasing in number in order to dampen inflammation and begin the repair process. We next investigated the specific role of M1 and M2 macrophages and their role in the development and or suppression of inflammation by adoptively transferring bone marrow-derived macrophages that were induced to be M1 or M2 phenotype in-vitro (Figure S1A and B). M1 or M2 macrophages were adoptively transferred intratracheally into CP infected mice 7 days after infection. We observed that bacterial burden in the lungs was unchanged between the groups 14 days after infection (data not shown). The mice were sacrificed 35 days after infection and assessed for lung inflammation and pathology. Lungs from the mice that received M1 macrophages were significantly more severely inflamed and had increased numbers of lymphoid aggregates (iBALTs) when compared to the mice that received M2 macrophages (Figure 4B and C). Despite this severe inflammatory phenotype, the number of BAL cells remained the same (Figure 4D). Additionally, neutrophil and alveolar macrophage counts were also unchanged. However, there was a significant increase in the number of non-alveolar macrophages, along with increased monocytes, CD4, and CD8 T-cells in the lungs of mice that received adoptive M1 macrophage transfer (Figure 4D). The levels of IFN-γ, and IL-10, were unchanged between the groups, while there was significantly more IL-6 and IL-12p40 in the M1 adoptively transferred animals compared to the M2 group (Figure 4E). As there were more macrophages and monocytes in the M1 group, we checked the levels of MCP-1 in the lungs; while there was no significant difference, the M1 group was associated with a trend towards greater amounts of MCP-1 (Figure S2A).

### CP infection induces fibrosis and M1 macrophages enhances collagen deposition

**Figure 5:**
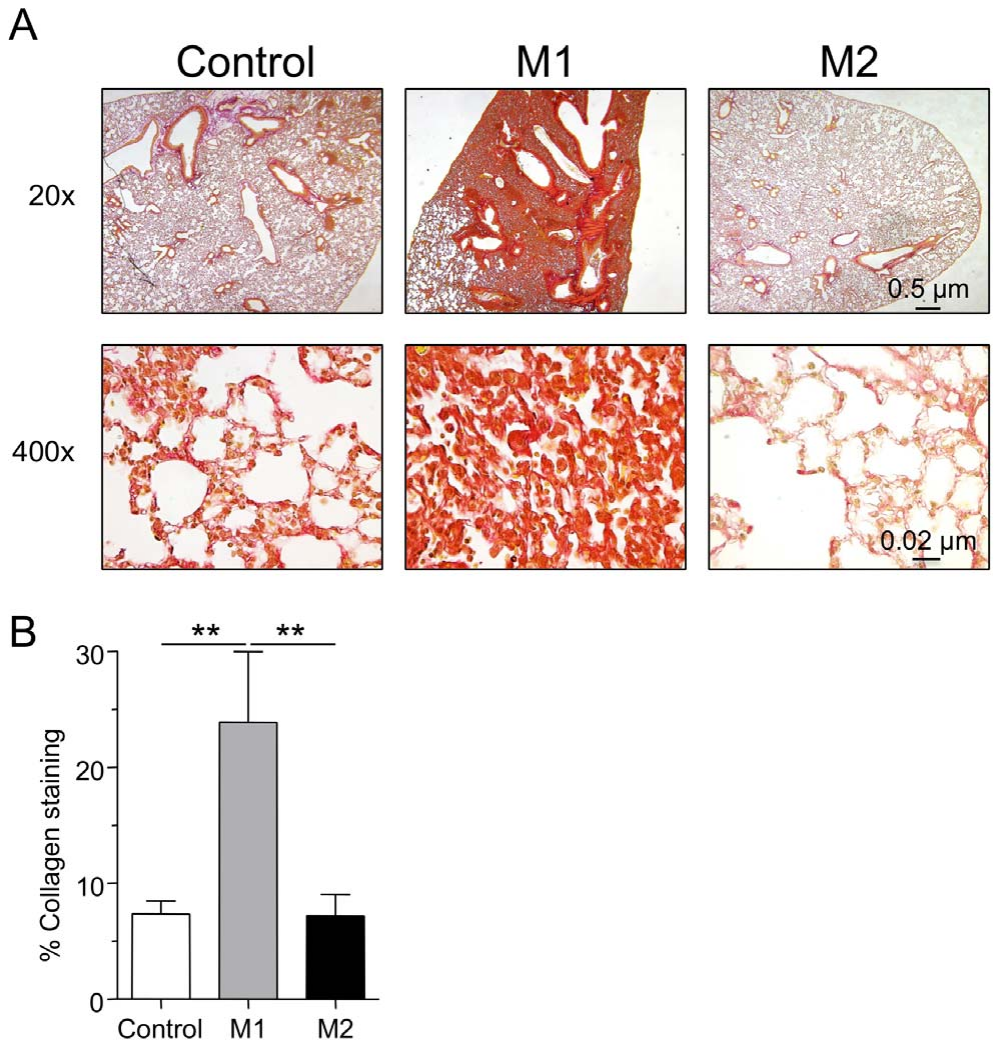
Adoptive transfer of M1 macrophages into CP infected mice induces severe fibrosis. A) Representative pico-sirius red stained lung sections. B) % collagen staining of lung sections. Data for all experiments shown represent at least two independent experiments (pooled together). *p<0.05, **p<0.01, ***p<0.001 (One-Way ANOVA).

We next assessed the presence of collagen as a marker for fibrosis 35 days after CP lung infection. We analyzed lung tissue sections using Pico-Sirius red staining and found collagen deposition in all tissue samples where CP infection had occurred (Figure 5A). Mice that had been infected with CP and allowed to run its course for 35 days had easily detectable collagen lining the alveoli and the larger airways. The lungs of mice that had received adoptive M1 macrophage transfer were intensely fibrotic while those from mice that received M2 macrophages were similar to control animals (Figure 5A). We observed a significant increase in lung collagen content in the M1 macrophage group compared with the control and M2 macrophage groups by collagen staining (Figure 5B). We also assayed for the amount of IL-17A and IL-23 in these lung tissues to see if these cytokines may be associated with the development of fibrosis, however no significant differences were found between the groups (Figure S3A), suggesting that IL-17A does not play a major role in the development of fibrosis in this experimental condition. Overall, these data suggest that excess M1 macrophages can drive inflammation, despite reduced bacterial IFUs, which in turn results in a long-term chronic inflammation with enhanced lung fibrosis and tissue damage.

## Discussion

*Chlamydia pneumoniae* is a common cause of acute respiratory infection and is estimated to cause 7% to 10% of community-acquired pneumonia among adults [1]. Most cases of pneumonia due to CP seem to be relatively mild and self-limited. However, *C. pneumoniae* respiratory infection has a gradual onset, and symptoms may be of prolonged duration, despite antibiotic therapy. The immune response to this primary infection is at best partially protective, and reinfections are common [1], [30]. Like other chlamydial organisms, *C. pneumoniae* has a tendency to persist in tissues [1], [30]. Chronic *C. pneumoniae* respiratory infections have been associated with the induction or the acceleration of various inflammatory diseases such as chronic lung diseases, asthma, atherosclerosis, and even lung cancer [31]–[35]. While, randomized controlled treatment trials among individuals with a history of atherosclerosis have generally not been positive, these studies do not disprove that *C. pneumoniae* infection can induce atherosclerosis as discussed in several reviews [35]–[37]. *C. pneumoniae* respiratory infection can cause exacerbation of asthma and COPD. Serological evidence of acute and chronic *C. pneumoniae* infection is found in one third of patients with chronic obstructive pulmonary disease (COPD) exacerbation, often together with other concurrent bacterial infection [6]. The mechanisms by which *C. pneumoniae* infection might contribute to the induction and or exacerbation of these chronic inflammatory diseases is not fully understood, and *C. pneumoniae* remains an important human pathogen. Here we found that *C. pneumoniae* infection in mice induces chronic inflammation, the formation of iBALTs, and also leads to collagen deposition in the lungs leading to fibrosis.

Macrophages play an important role in immune responses and surveillance as they produce cytokines to alert the host immune cells and can also be transformed phenotypically by tissue environment. It is widely acknowledged that at least two types of macrophages, with distinct phenotypes, are found in the process of infection and repair; classically activated or inflammatory macrophages (M1) and alternatively activated macrophages (M2) [23], [24]. In an inflammatory environment, Th1 and Th17 (inflammatory T cells) produce GM-CSF, which drives macrophage maturation. GM-CSF together with LPS induce inflammatory macrophages (M1) from bone-marrow-derived macrophages (BMDM), which produce high levels of iNOS, IL-12 and TNF-alpha [26]. In contrast, M2 macrophages can be generated *in vitro* by culturing BMDM with M-CSF together with IL-4. M2 macrophages predominantly produce higher concentrations of arginase-1 and IL-10, are immunoregulatory, and can promote wound healing and modify the extracellular matrix through the secretion of proteases and growth factors [29]. During infection and repair macrophages switch their phenotypes depending on the tissue environment [38] and this phenotypic plasticity in general can be both beneficial (wound repair), or lead to inappropriate host responses resulting in tissue damage that may lead to chronic pathologies. In this study, we found that an excess of M1 macrophages in *C. pneumoniae*-infected lungs results in a severe and prolonged inflammation that is characterized by greater iBALT formation and extensive lung fibrosis. However, while the exact mechanism that is driving this extensive chronic inflammation is currently unknown, CP titers were still just detectable in the lungs of some mice 35 days after infection. Others have shown that even in culture negative animals, cortisone treatment can lead to reactivation of CP infection, suggesting a possible continual source of proinflammatory signals [39], [40].

Pulmonary fibrosis can occur in a wide spectrum of diseases, in association with connective tissue diseases such as rheumatoid arthritis and systemic sclerosis, in response to chronic inflammation such as in hypersensitivity pneumonitis, or as part of an idiopathic lung disease such as sarcoidosis or idiopathic pulmonary fibrosis (IPF) [41]. In the most serious cases, lung transplantation may represent the only viable option for therapy [42]. Conceptually, IPF pathogenesis is now thought to be a primarily fibrotic process rather than an inflammatory one [43], although it is well-recognized that subsets of macrophages play a significant role in either limiting or enhancing lung fibrosis [44]–[46]. It is thought that wound healing programs that are normally induced after infection or injury to repair damage and restore organ functionality may be inappropriately activated and result in aberrant repair leading to tissue remodeling and fibrosis [47]. In our model investigating the long-term effects of an acute pulmonary *C. pneumoniae* infection in a mouse model, we observed collagen deposition after the normal course of infection. M2 macrophages have been associated with wound healing and fibrosis [48]–[50] and we did find a shift in the numbers of M1 macrophages to M2 macrophages as the *C. pneumoniae* lung infection progressed over time. Interestingly, when we adoptively transferred M1 or M2 macrophages into infected animals, it was the animals that received M1 macrophages that presented with severe prolonged inflammation and fibrosis in the lungs. This suggests that the M1 macrophages, which are part of the normal response to clear acute *C. pneumoniae* lung infection, result in an enhanced acute response when present in excess numbers, with greater inflammation, tissue injury, and severe fibrosis. IL-17 plays an important role in inducing lung fibrosis [51], [52], however, we did not find any differences in the levels of IL-17A or IL-23 in the lungs of the animals following M1 or M2 macrophage transfer. On the other hand, M2 macrophages, when transferred into infected animals during the acute phase of the infection, did not lead to an increase in inflammation, and if anything, trended towards a slight reduction in inflammation when compared to control animals (but not fibrosis). It is possible that these M2 macrophages helped institute the repair process ahead of schedule or dampened inflammation. M1/M2 macrophage polarization phenotypes are highly plastic to external signals, and in conditions where M1 macrophages may persist in excess in the lungs during the initial *C. pneumoniae* infection it is more likely that the infection may lead to chronic lung inflammation and fibrosis.

We also observed that *C. pneumoniae* infection induces iBALT formation in the lungs; to our knowledge, this represents the first such report for this pathogen. These secondary lymphoid structures are believed to play a role in local immunity by providing a rapid response to a repeat infection [16]. However, iBALTs have also been implicated in inducing ongoing lung pathologies, including in COPD, tuberculosis, and pulmonary complications of rheumatoid arthritis [17]-[19]. Considering that *C. pneumoniae* lung infection does not appear to provide lasting immunity [1], it seems likely that if iBALTs do play a protective role, their effects are minimal at best. In our model, the number of iBALTS correlated with the intensity of the inflammation. While iBALT formation has been linked to IL-17 [53], as discussed before, we did not see an increase in IL-17A or IL-23, suggesting another mechanism of development. However, this does not preclude a requirement for IL-17-producing cells in *C. pneumoniae* infection-induced iBALT formation. Other unknown aspects of the greater inflammatory response may drive the increase in iBALT numbers in the M1 adoptively transferred animals instead.

*Chlamydia pneumoniae* remains an important human pathogen, because it is ubiquitous and has been associated with many chronic inflammatory diseases. Here we show for the first time that *C. pneumoniae* can induce iBALT formations in the lungs, which may play a role in chronic lung pathology associated with this pathogen. We also showed the sequential move from M1 to M2 macrophages during the acute infection. Identification of putative tissue macrophage subsets is of great interest, because it is now believed that an imbalance of homeostatic (M2) versus inflammatory or pathogenic macrophage subsets (M1) may be driving the pathogenesis of inflammation and disease [24]. This study paves the way for elucidating the role of macrophage subpopulation in chronic lung disease pathogenesis and COPD associated with *C. pneumoniae* infection, and raises the possibility of selectively targeting inflammatory macrophage subpopulations to attenuate lung disease. Finally, as research into the genetic manipulation of Chlamydia is finally yielding tangible results [54], [55], advances into mechanistic insights related to host-pathogen interactions should be forthcoming.

## Materials and Methods

### Ethics statement

All animal experiments were performed according to the guidelines and approved protocol (IACUC Protocol #2097) of the Cedars-Sinai Medical Center Institutional Animal Care and Use Committee. Cedars-Sinai Medical Center is fully accredited by the Association for Assessment and Accreditation of Laboratory Animal Care (AAALAC International) and abides by all applicable laws governing the use of laboratory animals. Laboratory animals are maintained in accordance with the applicable portions of the Animal Welfare Act and the guidelines prescribed in the DHHS publication, Guide for the Care and Use of Laboratory Animals.

### Mice

6-7 wk old C57BL/6 mice (Jackson Laboratories, Bar Harbor, ME) were used throughout the study and were housed under specific pathogen-free conditions [56].

### Infection with C. pneumoniae

*Chlamydia pneumoniae* CM-1 (ATCC, Manassas, VA) was propagated in HEp-2 cells as described [57]. HEp-2 cells and *C. pneumoniae* stocks were determined to be free of Mycoplasma contamination by PCR. IFUs were assessed in Lung homogenates (suspended in 0.2 M sucrose, 0.02 M sodium phosphate (pH 7.2), 5 mM glutamate buffer) by propagation in Hep2 cells as before. Inclusions were counted using the Pathfinder Chlamydia Culture Confirmation System (BIO-RAD, Hercules, CA, USA) according the manufacturers protocol. Mice were intratracheally infected with 5×10^5^ IFU of *C. pneumoniae* in 50 µl of sterile PBS, while control mice received sterile PBS alone. Lungs were collected at various time points after infection and used for analysis of cell numbers, cytokines and bacterial numbers. In some experiments, 7 days after infection, 1×10^6^ macrophages (M1 or M2) were adoptively transferred into mice intratracheally followed by sacrifice 35 days post infection for analysis as above.

### Histopathological analysis

Lungs were fixed in 4% formalin, paraffin-embedded, sectioned at 0.5 micron thickness and stained with hematoxylin and eosin. The stained sections were then scored by a trained pathologist blinded to the groups of the experiment as previously described [11]. Briefly, the degree of inflammation was assigned an arbitrary score of 0 (normal  =  no inflammation), 1 (minimal  =  perivascular, peribronchial, or patchy interstitial inflammation involving less than 10% of lung volume), 2 (mild  =  perivascular, peribronchial, or patchy interstitial inflammation involving 10–20% of lung volume), 3 (moderate  =  perivascular, peribronchial, patchy interstitial, or diffuse inflammation involving 20–50% of lung volume), and 4 (severe  =  diffuse inflammation involving more than 50% of lung volume). Immunohistochemistry was performed using anti-CD3 (clone 2GV6) prediluted from Ventana Medical Systems (Yuscon, AZ), anti-CD20 (goat polyclonal, 1∶50) from Santa Cruz Biotechnologies (CA) and anti-CD21 (EP3093) for follicular dendritic cells (fDCs) from Cell Marque (Rocklin, Ca).

### Fibrosis analysis

Paraffin embedded lung sections were stained using the pico-sirius red staining method for collagen deposition. Analysis of collagen in stained slides was performed using the BZ II Analyzer software ver 2.1 (Keyence Corp. Japan). Briefly, a threshold level of detectable stain was assigned and the area of stain was measured and compared to the total lung area.

### Detection of chemokines and cytokines

The chemokine and cytokine concentrations in lung homogenates were determined using by Duoset Mouse MCP-1, MIP-2 (R&D systems, Minneapolis, MN, USA), OptiEIA Mouse IL-6 ELISA Set (BD Biosciences, San Jose, CA, USA) and Mouse IFN-γ, Mouse IL-10, Mouse IL-12p40, Mouse IL-23, and Mouse IL- 17A Elisa kits (eBioscience, San Diego, CA, USA). The assays were performed as described manufacturer protocol.

### Flow Cytometry

Total lung or Bronchoalveolar lavage (BALF) cells were collected at the indicated time points from mice infected with CP. Single cell suspensions were prepared from the lungs. Briefly, the lung cells were prepared by digesting the lungs with 0.1 mg/ml Liberase TM (Roche, Mannheim, Germany) and 50 units/ml of DNase I (Roche, Mannheim, Germany) and filtering through a 70 mm cell strainer (BD Biosciences). Erythrocytes were depleted by lysis buffer before staining. Isolated single cells were stained with following specific mAbs after Fc receptor blocking with CD16/32 Ab (clone 93). Numbers of various cell types were identified using F4/80 APC (clone BM8) and E450 CD11c (clone N418) for macrophages, CD11b Percp-CY5.5 (clone M1/70) and LY6G FITC (clone 1A8) for neutrophils, CD3 PE (clone 145-2C11) and CD4 Percp-Cy5.5 (clone RM4-5) for CD4+ and CD8 FITC (clone 53-6.7) CD8+ T cells. All antibodies were purchased from ebioscience (San, Diego, CA). RELM-alpha antibody (Abcam, MA) and anti-CD206 PE (R&D systems, Mn) was used to detect M2 macrophages. iNOS FITC antibody was used to identify M1 macrophage was purchased from ebioscience, San Diego, CA. For intracellular staining, cells were permeabilized using Cytofix/Cytoperm kit (BD Biosciences) and stained with mouse iNOS FITC or RELM-a. Flow cytometric analysis was performed using a CyAnTM flow cytometer (Beckman Coulter) and the data was analyzed using Summit (Dako, Carpinteria, CA, USA) software.

### Preparation of Bone marrow macrophages (BMDM) and *in vitro* stimulation

Femora and tibiae of 8–10 wk old C57BL/6 were flushed with RPMI 1640 medium. Bone marrow cells were cultured in RPMI1640 medium containing 10% FBS and 15% L929 cell conditioned medium. BMDM were harvested at day 7 and treated overnight with 40 ng/ml of IL-4 for differentiation to M2 macrophage or 40 ng/ml of IFN-γ for differentiation to M1 macrophage.

### Statistics

Data are reported as mean values±SEM. Statistical significance was evaluated by Student's *t* test and a *p* value <0.05 was considered significant. For multiple comparison test, statistical significance was evaluated by one-way ANOVA with Tukey's post-hoc test.

## Supporting Information

Figure S1 **M1 and M2 phenotype induction of bone marrow derived macrophages (BMM).** A and B) BMM were treated with IFN-γ or IL-4 over night and assessed for M1 or M2 skewing by A) iNOS production measured by ELISA and B) iNOS and CD206 staining using flow cytometry.
(PDF)

Figure S2 **M1 and M2 macrophage numbers after adoptive transfer of M1 and M2 macrophages.** A) M1 and M2 macrophage numbers in lung single cell suspensions after CP infection and adoptive transfer of M1 and M2 BMM. B) MCP-1 concentration in lung homogenates after CP infection and adoptive transfer of M1 and M2 BMM.
(PDF)

Figure S3 **IL-17A if not affected by macrophage adoptive transfer.** A) IL-17A and IL-23 concentration in lung homogenates after CP infection and adoptive transfer of M1 and M2 BMM.
(PDF)
