# Supplementary figures and images for: Chlamydia pneumoniae Infection in Mice Induces Chronic Lung Inflammation, iBALT Formation, and Fibrosis
Source: PLoS One. 2013 Oct 25;8(10):e77447. doi: 10.1371/journal.pone.0077447 (PMC3808399; doi:10.1371/journal.pone.0077447)

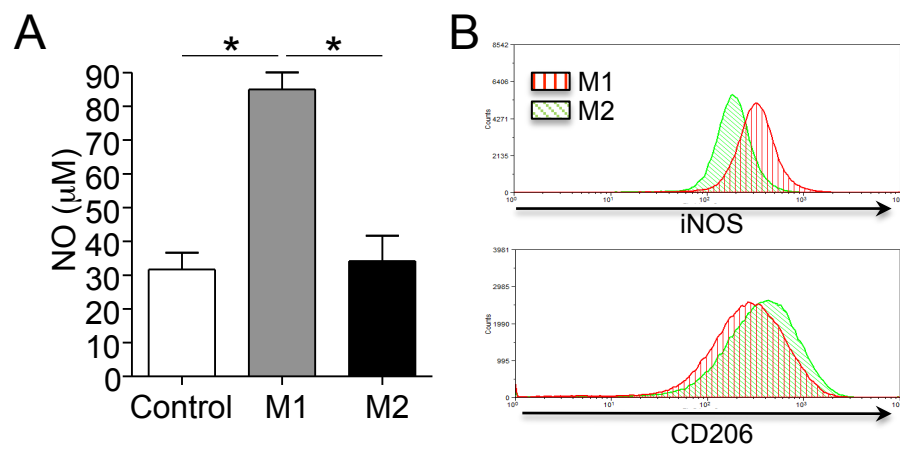

Figure S1

Supplement: Figure S1 — M1 and M2 phenotype induction of bone marrow derived macrophages (BMM). A and B) BMM were treated with IFN-γ or IL-4 over night and assessed for M1 or M2 skewing by A) iNOS production measured by ELISA and B) iNOS and CD206 staining using flow cytometry. (PDF) [file pone.0077447.s001.pdf]

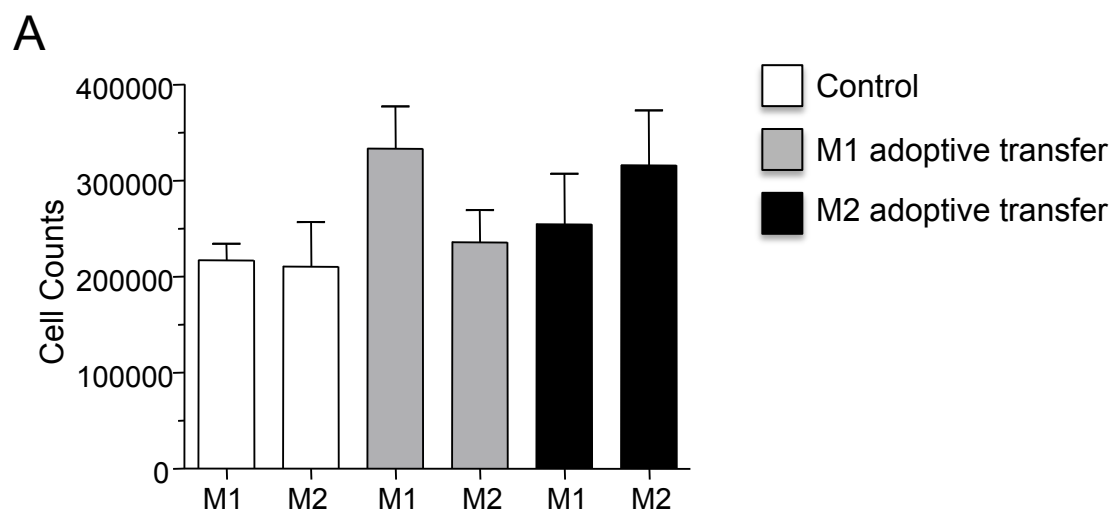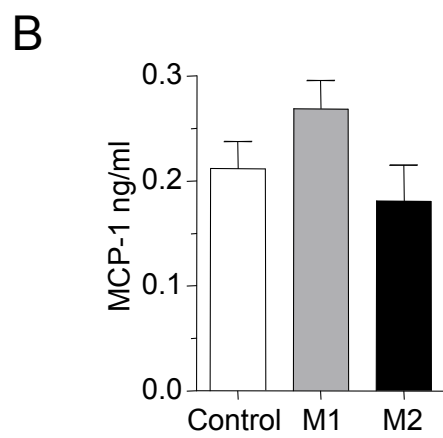

Figure S2

Supplement: Figure S2 — M1 and M2 macrophage numbers after adoptive transfer of M1 and M2 macrophages. A) M1 and M2 macrophage numbers in lung single cell suspensions after CP infection and adoptive transfer of M1 and M2 BMM. B) MCP-1 concentration in lung homogenates after CP infection and adoptive transfer of M1 and M2 BMM. (PDF) [file pone.0077447.s002.pdf]

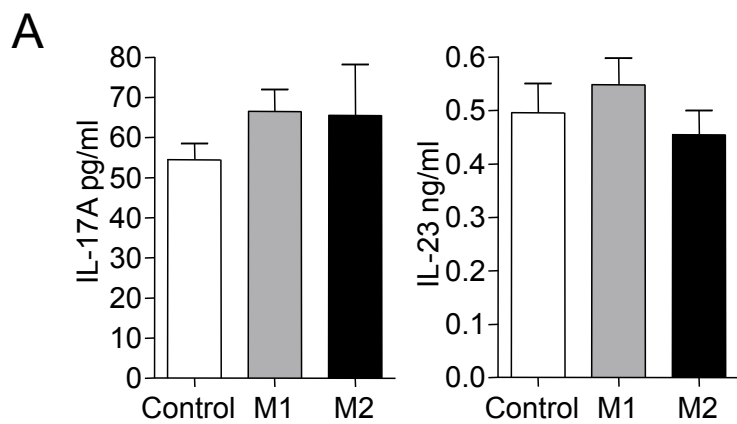

Figure S3

Supplement: Figure S3 — IL-17A if not affected by macrophage adoptive transfer. A) IL-17A and IL-23 concentration in lung homogenates after CP infection and adoptive transfer of M1 and M2 BMM. (PDF) [file pone.0077447.s003.pdf]
